# Rock crack initiation triggered by energy digestion

**DOI:** 10.1038/s41598-024-66226-3

**Published:** 2024-07-02

**Authors:** Lei Yan, Jian Chang, Ebelia Manda, Helin Li, Qian Wang, Yangfan Jing

**Affiliations:** 1 Sinosteel Maanshan General Institute of Mining Research Co., Ltd., Xitang Road 666, Maanshan, 243000 People’s Republic of China; 2 State Key Laboratory of Safety and Health for Metal Mine, Xitang Road 666, Maanshan, 243000 People’s Republic of China; 3 Mining Engineering Department, School of Mines and Mineral Sciences, Copperbelt University, 21692 Kitwe, Zambia

**Keywords:** Crack initiation, Energy digestion Index, Energy evolution, Rock strength, Rock mechanics, Natural hazards, Solid Earth sciences, Engineering

## Abstract

The critical value of rock failure is determined by irreversible deformation (inelastic deformation, damage, and other internal dissipation) processes and external conditions before rock failure. Nevertheless, a thorough explanation of the mechanism causing cracks in rock material has not yet been provided. The strain energy theory is applied in this work to assess the initiation of rock cracks and investigate the relationship between energy digestion and rock strength. Firstly, the uniaxial compression test was conducted on sandstone samples under quasi-static loading conditions and the results of energy evolution, non-linear cumulative digestion, and stored ultimate energy were obtained. Then, a novel algorithm for assessing the initiation of rock cracks has been put forth. The concept of energy digestion index (EDI), which is the ratio of digested energy over the external loading energy, has been developed to characterize the energy absorption capacity of rock material. The result shows a relationship between the maximum growth rate of energy digestion and the increasing rate of variable elasticity modulus and crack initiation. The mechanical characteristics and peak strength of the rock material are negatively correlated with the EDI. By monitoring the digested energy status, an evaluation of the residual strength is introduced based on the relationships, which will initiate further research into in-situ monitoring and failure prediction.

## Introduction

Disasters related to brittle fracture of rock occur frequently during the civil excavation process. Events such as rock bursts and slope landslides are often triggered by exceeding the ultimate strength of rock during tuning, sloping, mining excavation, or other civil constructions.

As a typical kind of heterogeneous natural body formed by mineral debris under geological action. Some internal elements of the rock material are damaged and fatigued as a response to external force, and a large number of initial micro-cracks form and grow, eventually leading to instability and failure. Many researches have been conducted on micromechanical damage fatigue based on laboratory experiments.

**Figure 1:**
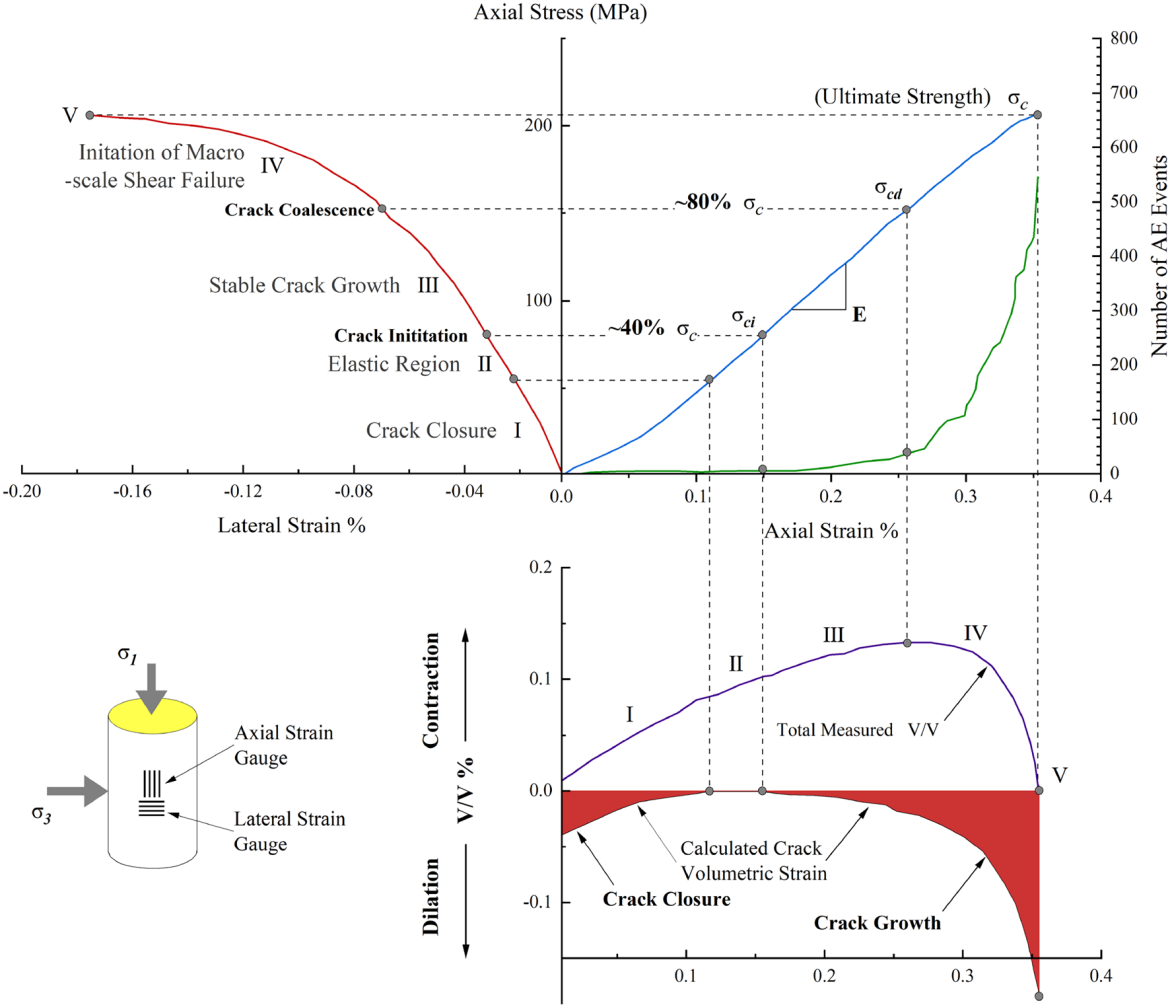
Stress–strain diagram showing the stages of crack development (Lac du Bonnet granite)^2–4^.

Griffith conducted a systematic study on stress and crack expansion when cracks occur in the internal structure of the material as early as 1920. The criterion for crack expansion under bidirectional compression was obtained by establishing a crack model for plane compression. Then, from the perspective of energy release, the energy balance theory, which is based on the ideal brittle material, is proposed, which differs from the traditional concept of material strength in the continuum^1^. CD Martin et al.^2–4^ found that the failure of brittle rocks can be classified into five stages based on the stress–strain curve: compaction closure stage, elastic deformation stage, stable crack expansion stage, accelerated crack expansion stage, and after the peak as showed in Fig. 1. Crack initiation was characterized as occurring at a threshold value of about 40% *σ*_c_. In advance of this, the material's elastic deformation and fracture closure stage match to the strain behavior. After the point of crack initiation, the rock material experiences stable cracking, and when stress levels reach around 80% *σ*_c_, macro-scale failure occurs.

Rock microscopic damage can now be directly observed using new technologies such as computerized tomography (CT) and nuclear magnetic resonance (NMR)^5–7^, In-situ monitoring of rock behavior remains difficult to achieve^8^. Indirect observation methods such as acoustic emission (AE), allow the passive monitoring of the elastic ultrasonic wave that propagates in the material as a result of the crack formation’s abrupt release of energy and the energy dissipation in the process of rock damage by studying the correlation between acoustic emission signals and mechanical properties of rock^9–13^, shown in Fig. 1. However, it is difficult to strictly correspond to the microscopic damage and reveal the process of damage evolution with non-destructive^14,15^and have not yet been universally formed for the most commonly used Prediction evaluation criteria method^16^.

More recently, the perspective of energy evolution has provided a new idea for the study of crack evolution. Shao et al.^17^ and Salari et al.^18^ proposed that the formation and development of cracks lead to dilatation, and the rock damage is characterized by volumetric strain energy. Further, the damage evolution can be characterized by elastic bulk modulus and plastic strain energy release rate^19–21^. A large number of laboratory experiments and theoretical analyses have confirmed the intrinsic relationship between strain energy dissipation and release in the process of rock deformation and failure^20,22–25^. Based on the above findings, the stress–strain curve of hysteresis loops obtained by uniaxial compression has been used to characterize the strain energy. However, due to the energy dissipation caused by compaction and plastic damage at an uncertainty of the stage of occurrence^26^, the hysteresis curve cannot be identified; other side, the process of energy transformation has the characteristics of self-inhibition, bifurcation, and chaos, and the energy accumulation is iterative growth^27^. Consequently, the accumulated elastic energy calculated by the hysteresis curve has a large deviation.

These work and theoretical research results have fully demonstrated the energy evolution characteristics of rock under load, which has laid a foundation for theoretical research in the laboratory, but it is still not practical in engineering in-situ monitoring and disaster prediction.

In this study, we aimed to overcome these limitations by using the energy digestion index (EDI) a novel algorithm, based on energy theory to determine the trigger point of intact rock crack initiation to investigate the relationship between rock strength and energy digestion. Through laboratory rock mechanics tests and the energy accumulation process of loading samples, the results show that once the increased rate of energy digestion reaches its maximum, rock crack initiation occurs. By using EDI, we were able to accurately model the relationship between rock strength and energy digestion, leading to a more comprehensive understanding and exploration of In-situ monitoring of rock damage and failure prediction.

## Energy evolution characteristics

### Energy conversion analysis

According to the principle of conservation of energy, the process of external work on the sample is accompanied by energy conversion; when the material is an ideal brittle rock, the external work is completely converted into elastic strain energy and stored within the material. The digested energy is to overcome the rock's intrinsic cohesion in the phase of crack evolution, which means there is no extra energy dissipation until rock strength reaches the peak^10^.

Due to the heterogeneity of rock materials, not all external work is converted into elastic strain energy and stored inside the material, and it is accompanied by other forms of energy dissipation, such as elastic strain energy^28^, acoustic emission^29–31^ that releases energy in the form of elastic waves phenomenon: deformation energy^32^, which causes the internal state of the material to change, thermal energy^33^, and so on. This portion of the energy is primarily used to reconstruct the internal stress field distribution, as well as to generate and decimate micro-cracks, which have been observed by the acoustic emission study of rock specimens under uniaxial loading^23,26,29–32,34,35^.

The uniaxial compression process is accompanied by a certain level of AE ringing count events before approximately 50% of compressive strength, and the occurrence of the events is random^10^. Xie Heping^23^ assumed that there is no heat exchange between the rock and the outside in the process, according to the first law of thermodynamics, its form is given in the following:

1 $$ U = U^{e} + U^{d} $$

2 $$ U^{e} = \frac{1}{2}(\sigma_{1} \varepsilon_{1} + \sigma_{2} \varepsilon_{2} + \sigma_{3} \varepsilon_{3} ) = \frac{1}{2E}[\sigma_{1}^{2} + \sigma_{2}^{2} + \sigma_{3}^{2} - 2\nu (\sigma_{1} \, \sigma_{2} + \sigma_{1} \sigma_{3} + \sigma_{3} \sigma_{2} )] $$

where *U* is the total energy density of external workmanship, *U*^*e*^ is the elastic strain energy density, and* U*^*d*^ is the dissipated energy density.

**Figure 2:**
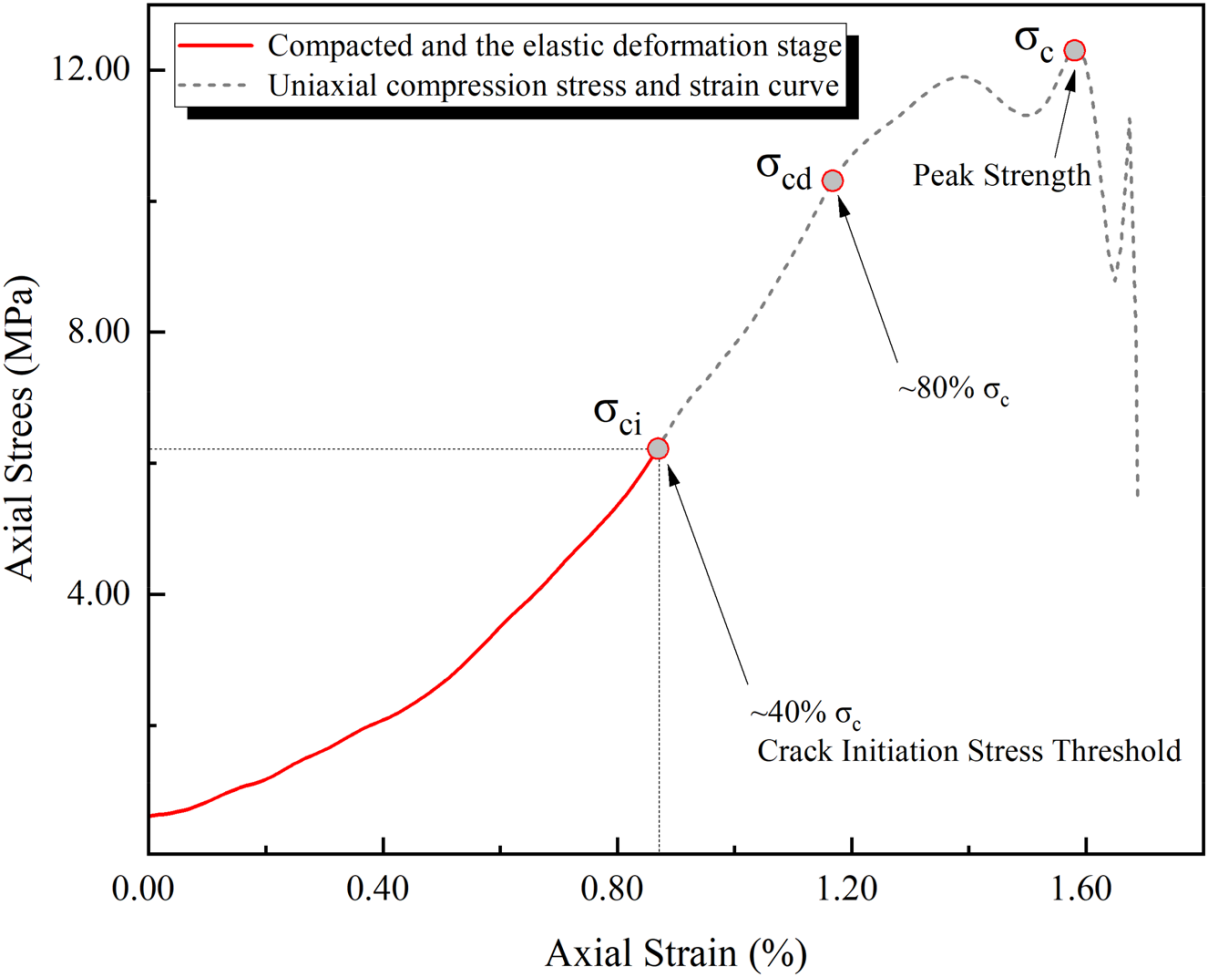
The uniaxial compressing stress–strain curve schematic diagram.

The one characteristic curve of uniaxial compression stress and strain obtained, shown in Fig. 2, is used as an example to illustrate the energy evolution of the compacted and the elastic deformation stage before the crack initiation stress threshold.

### Measures of elastic strain energy density

Four assumptions are considered in this paper, that: (i) the rock material is perfectly elastic (no energy dissipation in elastic deformation and all elastic strain energy released after force removed) ; (ii) define the unit volume *V* and the unit has a closed surface *S*, $\delta W$ and $\delta U$ are the work of the external force and the increment of internal energy for unit volume, respectively; (iii) the external force is applied constantly, regardless of the effect of strain rate, to ensure that the unit body is in equilibrium at any time; (iv) the kinetic energy changes are ignored; These are guaranteed to be a quasi-static loading in uniaxial compression.

Based on the above assumptions, the stress tensor is decomposed into spherical and deviatoric stress tensors; the strain tensor is decomposed into spherical and deviatoric strain tensors, that is, strain energy is decomposed into the energy of volume $W_{1}$ and the energy of shape change $W_{2}$, the generalized form given in the following, respectively.

3 $$ W_{1} = \iiint_{V} {\left( {X_{u} + Y_{v} + Z_{w} } \right)}dV $$

4 $$ W_{2} = \iint_{S} {\left( {X^{*} u + Y^{*} v + Z^{*} w} \right)dS} $$

where $X$, $Y$, $Z$ defined as the external force on per unit volume element, $X^{*}$, $Y^{*}$, $Z^{*}$ are the internal force on the surface of the unit element, the external force work *W* is entirely transformed into elastic strain energy and is defined as:

5 $$ W = W_{1} + W_{2} = \iiint_{V} {\left( {X_{u} + Y_{v} + Z_{w} } \right)}dV + \iint_{S} {\left( {X^{*} u + Y^{*} v + Z^{*} w} \right)dS} $$

The work done by the external force acting on the unit body to produce a small displacement $\delta W$, is expressed as:

6 $$ \delta W = \delta W_{1} + \delta W_{2} = \iiint_{V} {X_{i} }\delta u_{i} dV + \iint_{S} {X_{i}^{*} }\delta u_{i} dS $$

After substituting into the balance equation and boundary conditions, it is known from the divergence theorem:

7 $$ \delta W = \iiint_{V} {\sigma_{ij} }\delta u_{i,j} dV $$

The increase of internal energy is found:

8 $$ \delta U = \iiint_{V} {\sigma_{ij} }\delta \varepsilon_{i,j} dV $$

Known from Green’s formula, and let:

9 $$ \frac{{\partial u_{0} \left( {\varepsilon_{ij} } \right)}}{{\partial \varepsilon_{ij} }} = \sigma_{ij} $$

Integrate the formula can easily find:

10 $$ \int_{0}^{{u_{0} \left( {\varepsilon_{ij} } \right)}} {du_{0} } = u_{0} \left( {\varepsilon_{ij} } \right) - u_{0} \left( 0 \right) $$

where $u_{0} \left( {\varepsilon_{ij} } \right),u_{0} \left( 0 \right)$ respectively indicate the elastic strain energy density after loading and before loading.

Let $u_{0} \left( 0 \right) = 0$, and the strain energy density is determined by its stress and strain increase, which can be defined as:

11 $$ u_{0} (\varepsilon_{ij} ) = \int_{0}^{{\varepsilon_{ij} }} {\sigma_{ij} } d\varepsilon_{ij} $$

The unit volume strain energy density of a rock sample can be expressed as:

12 $$ \frac{dW}{{dV}} = \int_{0}^{{\varepsilon_{ij} }} {\sigma_{ij} } d\varepsilon_{ij} $$

### Characteristics of strain energy evolution and initiation energy

According to Qingbin Meng et al.^28^ energy self-inhibition evolution model and Zheng Zaisheng's^36^ research on the nonlinear fitting formula of energy transfer in rock deformation under uniaxial compression with a certain stress level $\sigma$. The rate of increment of the accumulated strain energy density was given^27,34,37^ by:

13 $$ \frac{1}{{u^{i} }} \cdot \frac{{du^{i} }}{d\sigma } = a_{i} (u^{ci} - u^{io} ) - b_{i} u^{i} $$

The formula $u^{ci}$ represents the energy density converted from external input driving energy, $u^{i0}$ is the lowest input strain energy density threshold, and, $a_{i}$, $b_{i}$ is the coefficient, which respectively reflects the degree of energy conversion and inhibition.

Integrate $u^{i}$ and find:

14 $$ u^{i} = \frac{{a_{i} \left( {u^{ci} - u^{i0} } \right)e^{{a_{i} \left( {u^{ci} + u^{i0} } \right)c/b_{i} }} e^{{a_{i} \left( {u^{ci} + u^{i0} } \right)\sigma }} }}{{c_{1} b_{i} \left[ {1 + e^{{a_{i} \left( {u^{ci} + u^{i0} } \right)c/b_{i} }} e^{{a_{i} \left( {u^{ci} + u^{i0} } \right)\sigma }} } \right]}} $$

with $c_{1}$, $c$ the integral constant.

Based on elasticity modulus $E_{i}$ and formula Eq. (13), find:

15 $$ E_{i} = \frac{{\sigma^{2} }}{{u^{i} }} $$

Derivative and let the first derivative be 0 as follows:

16 $$ \frac{{dE_{i} }}{d\sigma } = \frac{{2\sigma u^{i} - \left( {u^{i} } \right)^{{^{\prime } }} \sigma^{2} }}{{\left( {u^{i} } \right)^{2} }} = 0 $$

Equation (14) substituted and organized, can find:

17 $$ \sigma_{i} = \frac{2}{{a_{i} \left( {u^{ci} - u^{i0} } \right) - b_{i} u^{i} }} = \frac{2}{{a_{i} \left( {u^{ci} - u^{i0} } \right) - \frac{{a_{i} \left( {u^{ci} - u^{i0} } \right)e^{{a_{i} \left( {u^{ci} + u^{i0} } \right)c/b_{i} }} e^{{a_{i} \left( {u^{ci} + u^{i0} } \right)\sigma_{i} }} }}{{1 + e^{{a_{i} \left( {u^{ci} + u^{i0} } \right)c/b_{i} }} e^{{a_{i} \left( {u^{ci} + u^{i0} } \right)\sigma_{i} }} }}}} = \frac{{2a_{i} \left( {u^{ci} - u^{i0} } \right)}}{{1 + e^{{a_{i} \left( {u^{ci} + u^{i0} } \right)c/b_{i} }} e^{{a_{i} \left( {u^{ci} + u^{i0} } \right)\sigma_{i} }} }} $$

After being simplified, when the elastic modulus reaches the maximum value, the strain energy accumulated $u_{i}$ and Stress $\sigma_{i}$ are obtained:

18 $$ u_{i} = ce^{{ - \frac{1}{2}}} \sigma_{i} $$

And

19 $$ \sigma_{i} = \frac{{2a_{i} \left( {u^{ci} - u^{i0} } \right)}}{{1 + e^{{a_{i} \left( {u^{ci} + u^{i0} } \right)c/b_{i} }} e^{{a_{i} \left( {u^{ci} + u^{i0} } \right)\sigma_{i} }} }} $$

with $c$ the integral constant.

### Energy digestion index (EDI)

From the concept of strain energy density, with isothermal conditions, the unit volume strain energy density can be expressed as:

20 $$ \frac{dW}{{dV}} = \int_{0}^{{\varepsilon_{ij} }} {\sigma_{ij} } d\varepsilon_{ij} $$

**Figure 3:**
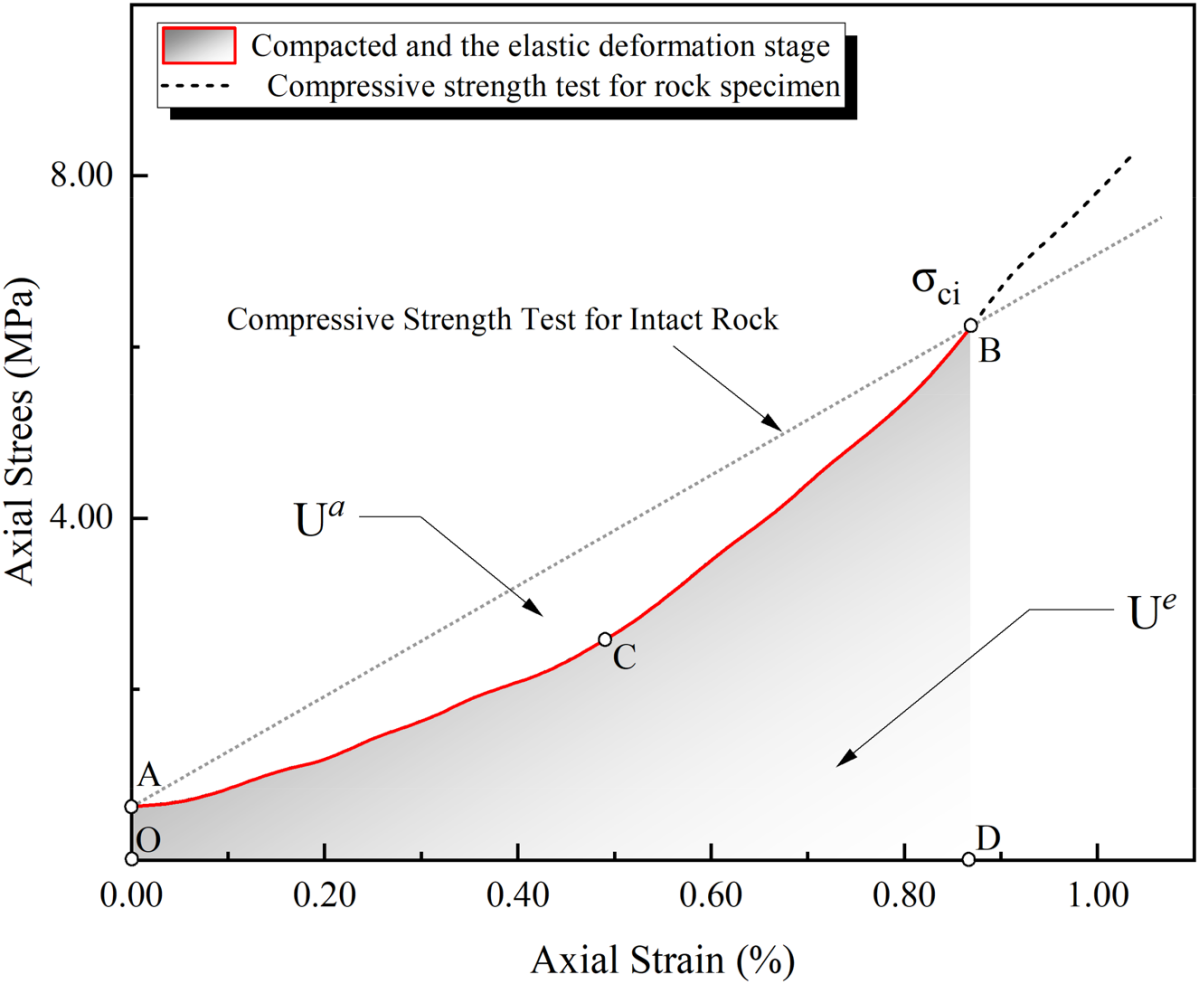
Schematic diagram of local amplification of stress–strain curves before crack initiation.

The above formula shows that the strain energy density of a unit can be determined by the stress and strain of the unit. The elastic stage of the stress–strain curve obtained from the experiment is partially enlarged, as shown in Fig. 3.

When the rock sample is loaded to point B in the uniaxial compression test, the elastic strain reaches a maximum, and the rock cracks. The area AOBD can be used to express the external force input energy during this process. The sample material is generally thought to be in the elastic stage at this point^35,38,39^. When the load is unloaded at point B, the formed unloading curve essentially coincides with the loading curve, indicating that no new crack damage is generated as a result of external force and no energy is dissipated^40–42^.

The strain energy digested by the rock sample is represented by the area AODBC. Area ACB represents the portion of the external force's work that has not been converted into strain energy. At this point, the energy $U$ generated by the external force can be divided into two parts: the strain energy $U^{e}$ converted and digested by the rock sample, and the energy loss $U^{a}$ the external force work cannot be converted into strain energy. The rock material's energy digestion index Eq. (20) is established by analyzing the proportion of the energy digested in the total input energy. This formula is used to characterize the degree of response of the rock material to external loads and reflect the properties of the material itself. The energy digestion index EDI can be expressed as:

21 $$ D = \frac{{U^{e} }}{U} = \frac{{U^{e} }}{{U^{a} + U^{e} }} \times 100\% $$

The above formula shows that when a rock material is subjected to an external load, the energy generated by the external force is not completely converted into elastic strain energy and stored inside the material due to differences in the rock material with microstructure and ability to withstand the outside; in this process, the mechanical properties of rock materials are characterized by the energy conversion and digestion ability.

## Compressive strength tests

### Specimens preparation

**Figure 4:**
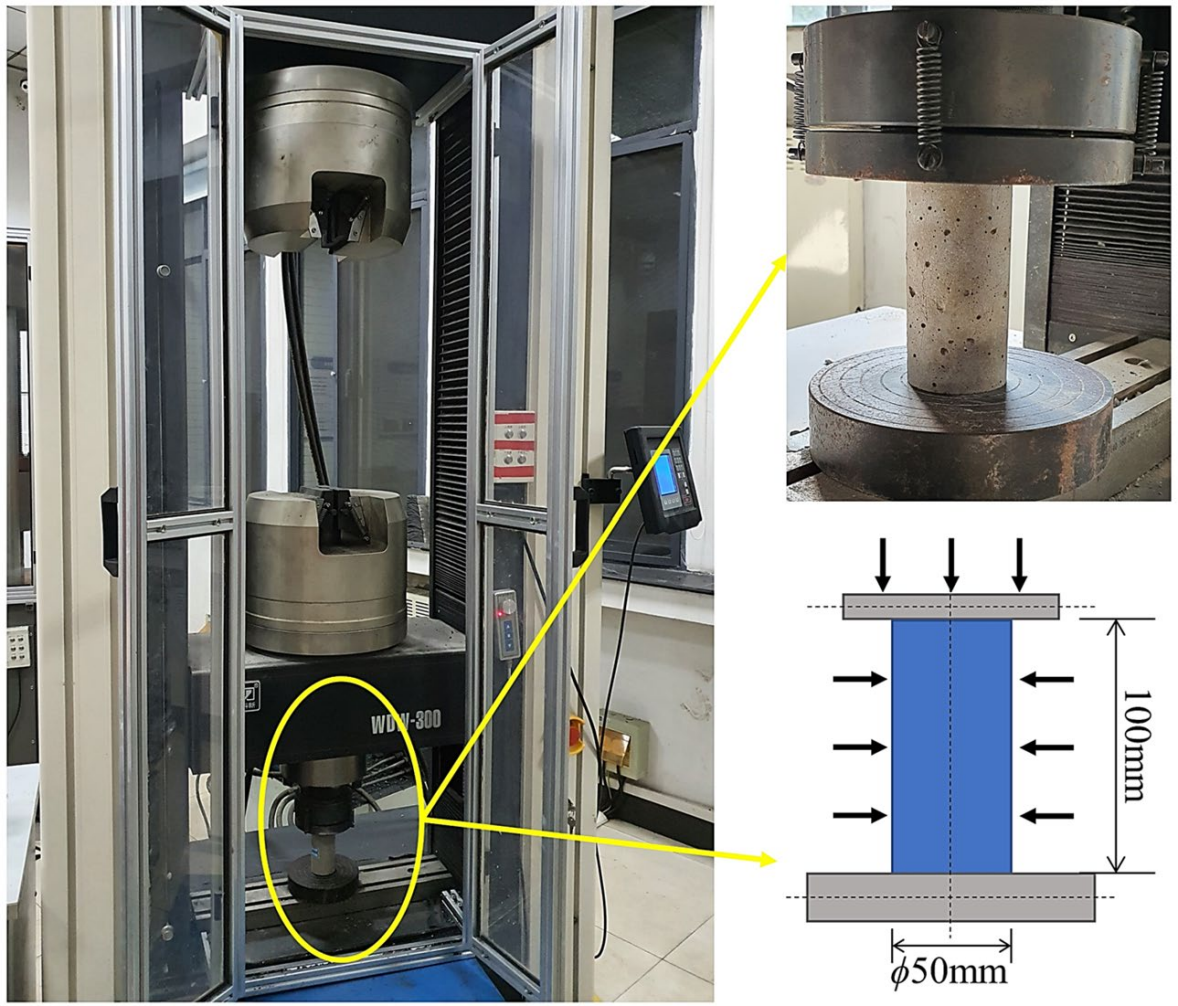
Compressive strength test for specimens.

All of the rock specimens examined were obtained from a Chinese coal mine. Sandstones with varying proportions of fine sandstone and siltstone as the main constituents exist. The specimens were processed into cylinders with a diameter of 50 mm and a length-to-diameter ratio of 2 (Fig. 4) using the ISRM-recommended method^43^. After careful grinding with a grinder and sandpaper, the top and bottom surfaces have flatness of less than 0.02 mm and parallelism of less than 0.05 mm. 9 specimens were tested due to the discreteness of the test data.

### Laboratory uniaxial compression testing

The tests were carried out using the WDW-300 digital electro-hydraulic servo system's equipment (Fig. 4) and the specimen was placed in the test machine. The loading rate of 0.0015 mm/s, with continuous loading until the specimen breaks. The test is terminated when the residual strength equals the 60% peak strength or maximum load 240 KN. In the test, 9 specimens were loaded uniaxially, and all the stress–strain data were recorded.

## Result and discussion

### The result of compressive strength test

**Figure 5:**
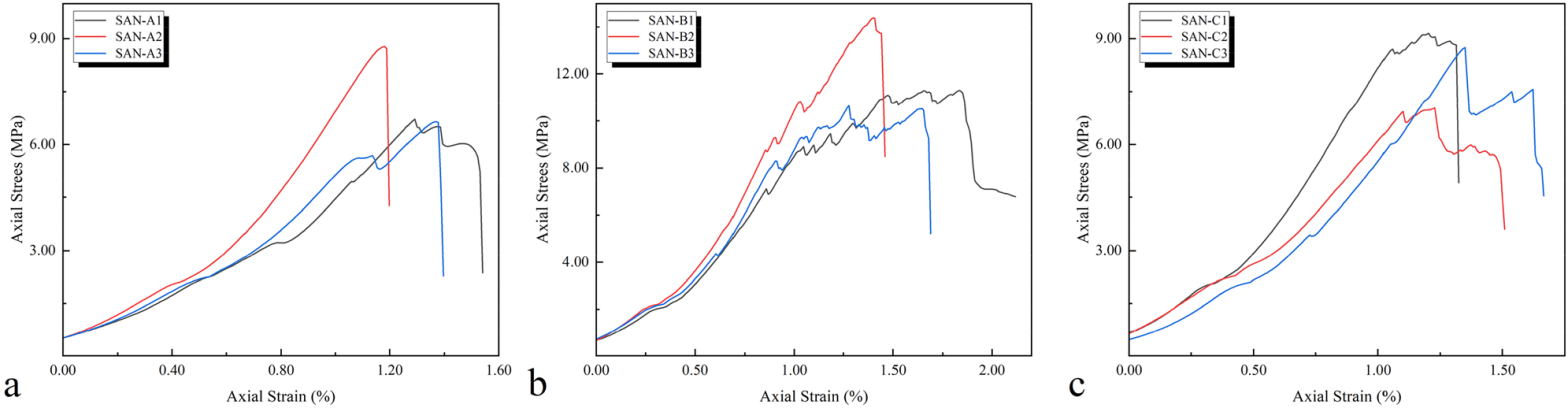
Stress–strain curve test results of specimens: (**a**) SAN-A (**b**) SAN-B (**c**) SAN-C.

Axial stress–strain curves for the three kinds of rock specimens under uniaxial compression are shown in Fig. 5, which are similar in geometry, and the axial stress-axial strain behavior can be approximately divided into four typical stages, i.e., fissure closure, elastic deformation, crack growth and propagation, and strain-softening. In the process of uniaxial compression of the specimen, the stress–strain characteristics obtained are similar to those of C.D. Martin et al.^2–4^ in the stress–strain study of brittle rocks, including: the compaction closure stage, elastic deformation stage, stable crack expansion stage, crack accelerated expansion stage, and post-peak stage (shown in Fig. 1); the division of each stage of the failure process depends on 3 important stress thresholds: crack initiation $\delta_{ci}$,crack evolution $\delta_{cd}$,ultimate strength $\delta_{c}$.

When the load is lower, the internal pores and cracks of the rock are compressed and sealed, and the process of the rock with better compactness is not obvious. The elastic properties of the rock body conform to Hooke's law, and the load reaches the threshold stress, which is 30–50% of the peak strength^4^.

Continue to load and enter the stage of stable crack propagation. After reaching the threshold stress that is 70–80% of the peak strength, the crack enters rapid and unstable expansion, macroscopic shear failure occurs, and finally reaches the peak strength. The process of fissure compaction and closure exists in every test, but it is not significant. The reason is due to the heterogeneity of the natural rock sample itself and the existence of inevitable internal defects and fissures. The original fissures and fissures in this stage defects are further compacted, and the volume of the original crack defects decreases and tends to zero, and enters the elastic stage; in the elastic stage, some curves show an almost completely elastic state, and the stress–strain curves show linear elastic characteristics; while most of the curves are almost completely elastic. It appears as a downward concave phenomenon, that is, the slope increases as the load continues, the elastic deformation exhibits nonlinear characteristics, and the one-to-one correspondence between stress and strain is still maintained:

22 $$ \sigma = E \cdot \varepsilon = f\left( \varepsilon \right) $$

**Figure 6:**
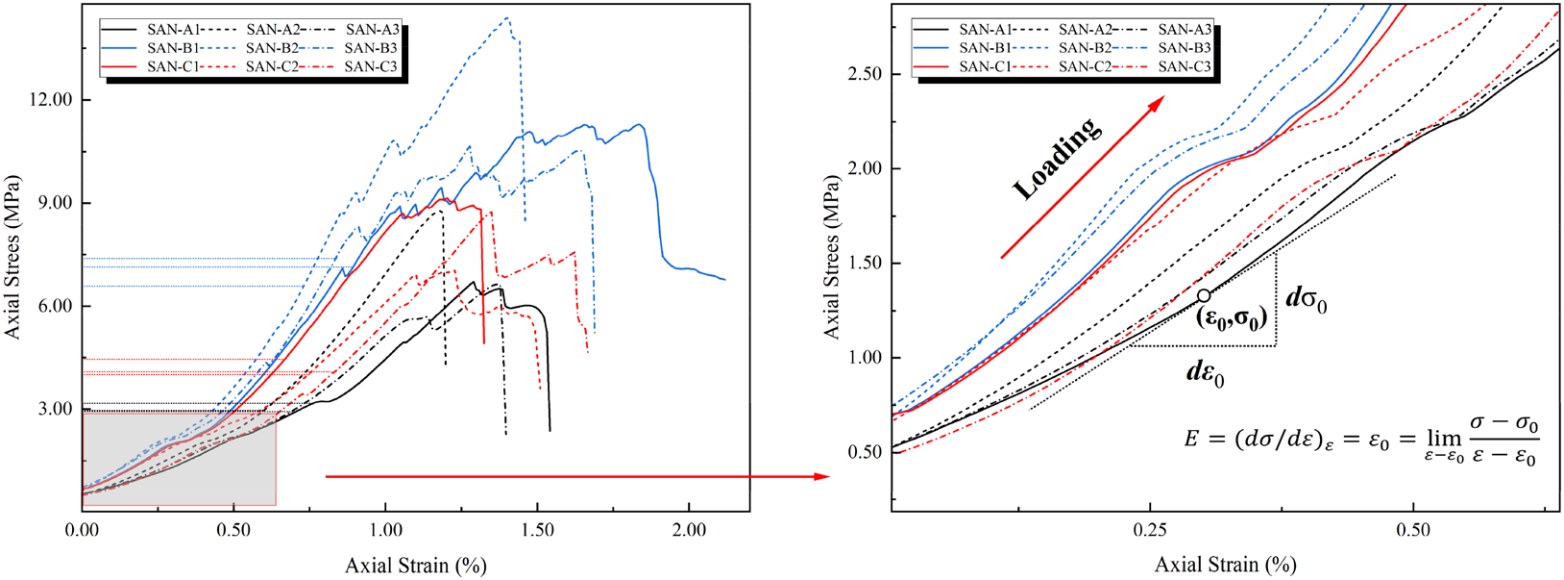
Differences in the end stage of elastic deformation of each specimen.

At the stage of fissure closure, the uniaxial compression stress–strain curve of the sample shows the downward concave and the initial nonlinear deformation at low-stress levels, which results from the closure of some primary pores and voids in the sample with the increasing compression stress. At the stage of elastic deformation, the loading begins with the increase of axial stress after the primary fissure closure, and the elastic deformation dominates the stress–strain curve of the sample. However, the slope of the three curves, i.e., the elastic modulus, has a big difference, which results from the component of the tested rock material. The rigidity of granite samples is the largest, and that of sandstone samples is the smallest. At the stage of crack growth and propagation, the sample continuously produces the stress concentration near the tips of the initial crack, which results in the initiation and propagation of some new cracks. Therefore, the stress–strain curve departs from the elastic behavior and shows distinctly nonlinear deformation. For granite and limestone samples, this stage is short, while it is long for sandstone samples. At the stage of strain-softening, the macroscopic crack in the rock sample comes out rapidly and the post-peak behavior in the stress–strain curves shows a rapid drop. At this time, the sample can only support a lower axial stress, even though no axial stress. But the slope of the three curves, i.e., the softening modulus, in Fig. 6, differences in the end stage of elastic deformation of each specimen, and the post-elastic deformation curves of higher strength specimen are more abrupt than low strength ones.

### Modulus of elasticity

The modulus of elasticity is variable. The point elastic modulus and strain increase with the stress adding; it is different from the constant elastic modulus of completely elastic material, that is, under external load, the external work is not 100% energy transfer, part of the energy is lost, and it causes the specimen such internal structure changes, resulting in strain hardening^36^.

### Compressive strength and stiffness

Through the analysis of the stress–strain curves of different specimens, it is found that the initial stage of the test, that is, the pressure-tight and stage curves have good consistency, and there is no obvious sudden change, indicating that the integrity of the sample is better, and the original cracks are less, which meets the requirements of the sample. After the elastic deformation stage, the uniaxial compression stress–strain curves of different specimens have obvious differences, and the material properties have changed significantly, as shown in Fig. 6.

The three groups of stress–strain curves all show variable elastic modulus in the pre-peak stage and the characteristics of failure after the sample reaches the ultimate strength as the external load continues to increase and there is a significant difference in strength. Compared with the SAN-B group, the SAN-A and SAN-C groups are more sensitive to the external load due to the smaller stress and strain under the action of the external load.

The mechanical properties of different sandstone specimens are shown in Table 1. It can be seen that the strength of different sandstone specimens under uniaxial compression is $6.7 - 12.1$ MPa. According to the rock's Protodyakonov index *F*, the specimens are divided into three groups: SAN-A, SAN-B, and SAN-C, with three specimens in each group, and identified in numbers 1, 2, and 3. The average of group SAN-A $F = 0.7$, the average of group SAN-B $F = 1.2$, and the average of group SAN-C $F = 0.8$.

**Table 1:** The result of compressive strength test and dispersion.

Specimens	Serial number	Cracking threshold	Avg. value	Ultimate strength	Avg. ultimate strength	Avg. density	Modulus of elasticity)	Protodyakonov coefficient
		σ_ci_/MPa	MPa	σ_c_/MPa	MPa	kg/m^3^	E_c_/MPa×10^2^	*F*
SAN-A	1	3.168	3.02	6.710	7.37	1941.64	4.00	0.70
	2	2.956		8.780			4.94	
	3	2.926		6.630			4.26	
SAN-B	1	7.139	7.03	11.30	12.11	2156.38	8.03	1.20
	2	6.579		14.39			9.01	
	3	7.376		10.65			8.78	
SAN-C	1	4.452	4.18	9.140	8.30	2011.37	6.65	0.80
	2	4.005		7.030			5.33	
	3	4.085		8.740			4.95	

### Energy digestion index (EDI)

According to the grouping of specimens with different Protodyakonov coefficients, the energy digestion index, sample cracking stress threshold, cracking energy threshold, and uniaxial compressive strength data of each specimen can be calculated, as shown in Table 2.

**Table 2:** Samples of EDI and energy intensity value.

Specimens	Serials number	EDI	Cracking stress threshold	Cracking strain energy threshold	Cracking strain	Ultimate strength	Cracking threshold/ultimate strength
		D/%	/MPa	/MJ·m^−3^	/%	/MPa	/%
SAN-A	1	92.643	3.168	1.297	0.76	6.710	47.21
	2	92.704	2.956	0.9669	0.6	8.780	33.67
	3	95.357	2.926	1.206	0.7	6.630	44.13
SAN-B	1	82.697	7.139	2.882	0.89	11.300	63.18
	2	81.075	6.579	2.142	0.73	14.390	45.72
	3	78.924	7.376	2.685	0.84	10.650	69.26
SAN-C	1	85.159	4.452	1.469	0.67	9.140	48.71
	2	93.524	4.005	1.632	0.75	7.030	56.97
	3	88.448	4.085	1.715	0.85	8.740	46.74

From Table 2, in the pre-cracking stage of the specimens with different Protodyakonov coefficients, the energy digestion index is relatively concentrated and the overall level is 78–95% higher, the ultimate strength during a failure is relatively discrete, ranging from 6.71 to 14.39 MPa; Group SAN-A samples average Protodyakonov coefficient *F* = 0.7, average energy digestion index D = 93.57%; SAN-B group average Protodyakonov coefficient *F* = 1.2, average energy digestion index D = 80.90%; SAN-C group average Protodyakonov coefficient *F* = 0.8, average energy digestion index D = 89.04%. It can be seen that the higher the EDI of the specimens, the lower the average hardness and the lower the ultimate strength.

**Figure 7:**
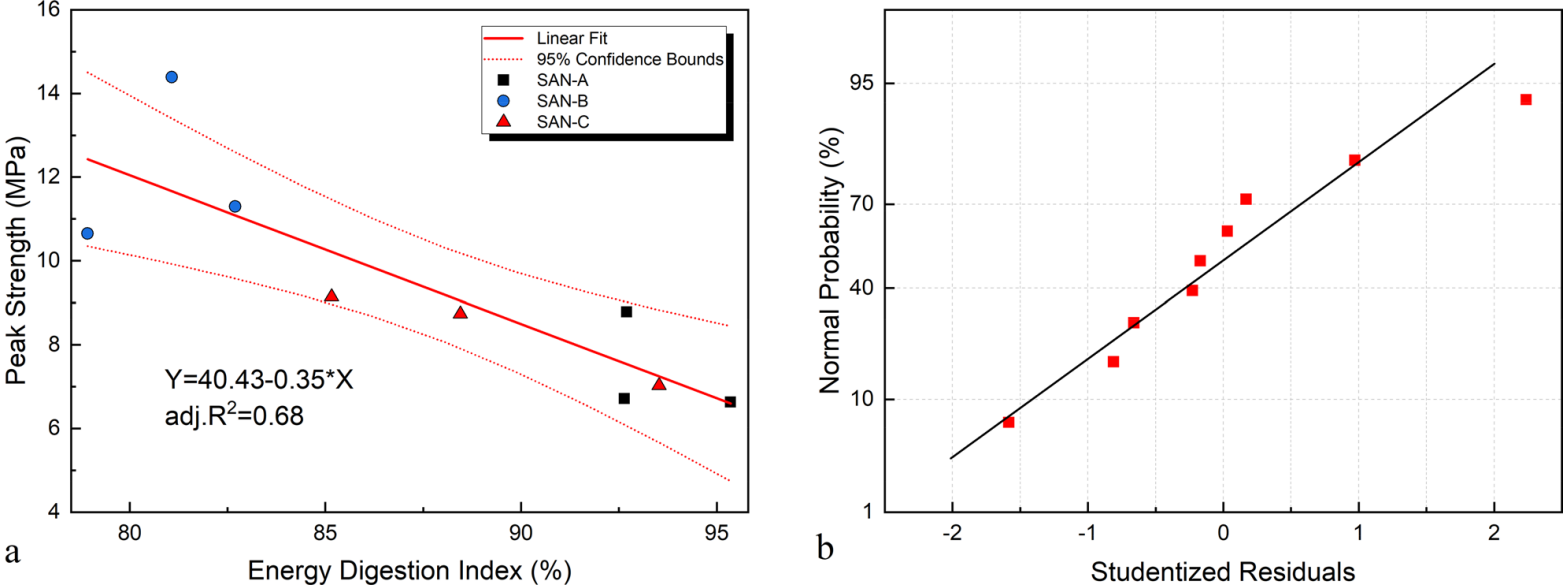
Model fitting: (**a**) fitting result of strength and energy digestion index (**b**) studentized residual error.

To investigate the relationship between energy digestion characteristics, rock strength, and sensitivity to external forces, the EDI and uniaxial compressive strength of each sample were tested under quasi-static loading conditions^38^. The EDI and ultimate strength data are shown in a rectangular coordinate system, and the results are fitted, as illustrated in Fig. 7.

In Fig. 7, the EDI of each rock sample and the uniaxial compression peak strength reflect the significant correlation between the strength of the rock sample and the energy digestion character. Concerning the law that the lower ultimate compressive strength with the higher the EDI in the elastic stage, the explanation for this phenomenon is that when the stress reaches the cracking stress, the energy obtained by the rock sample is all in the form of elastic strain energy accumulated in the material, and the internal damage can be ignored, the faster and more strain energy accumulates, the lower the strength of the rock, the easier it is to fail. The less the accumulated elastic strain energy, the poorer the rock's ability to digest introduced energy, and the rock has better strength characteristics because large-scale crack formation and destruction require more energy.

When the EDI reaches the ultimate value, the rock cracks sharply, and the elastic strain energy accumulated in the rock material is suddenly released in a short time, accompanied by macroscopic manifestations such as acoustic emission and debris ejection.

## Conclusions

Using the uniaxial compression test, stress–strain curve analysis, and energy calculation analysis of a specific mine sandstone sample, this article comes to the following results:

The test results for each sample clearly show a linear relationship between the fissure initiation threshold stress, expansion threshold stress, and peak strength; however, because each sample is unique, there are differences in the length of the strain process experienced at different stages.

The strain energy absorbed by the rock sample per unit volume grows throughout the elastic deformation stage as the external load work increases continuously, exhibiting nonlinear growth features. First, there is an increase in strain energy digestion, followed by a drop. This phenomenon demonstrates how the rock sample material's capacity to absorb and store elastic strain energy is reflected in how incompletely the work produced by external forces is converted into strain energy.

The article's suggested dimensionless parameter of the strain energy digest index in the elastic stage has a strong linear correlation with the rock sample's peak compressive strength. Additionally, it is evident from fitting that there is a clear linear relationship between the sample material and the strain energy that the rock sample digests during the early elastic deformation stage of uniaxial compression. The intensity increases with less strain energy being digested.

Lastly, it must be clarified that full compaction and the absence of natural fissures cannot be guaranteed by the test volume and sample material. The article's conceptions and findings, which mostly highlight the fact that typical rock materials frequently have a linear elastic stage, are still preliminary. The primary cause of the observed nonlinearity in the measurement is the loss of entropy; however, quantifying the energy lost in this process is a challenging task due to its complexity. The energy difference between the beginning of the elastic phase and the end indicates the energy dissipation and loss. Second, the findings of the experimental investigation offer insightful information about the strength and deformation properties of rock materials, and they examine the impact of these properties on their uniaxial strength.

To give further references for stability evaluation, the follow-up work will examine the energy digestion law of the in-situ rock mass in comparison to the energy loss properties of the rock material and the energy discrimination criterion for strain energy digestion.

### The study's findings show

1. The material absorbs and accumulates strain energy under the influence of external forces, displaying a non-linear accumulation in a changing modulus of elasticity.
2. The pace at which strain energy is absorbed by the rock material determines the creation of fractures, and when this rate of growth in strain energy digestion achieves its maximum, the material's growing rate of variable elasticity modulus also reaches its maximum.
3. The mechanical qualities of the rock material are inversely correlated with the energy digestion index; that is, the lower the rock strength, the higher the energy digestion index of the rock material.
4. The correlation between the energy digestion index and the sensitivity of the rock material to external loading is not significant.

## Data Availability

All the data generated and/or analyzed during the current study is presented in the paper.
